# An exact analysis of unsteady MHD free convection flow of some nanofluids with ramped wall velocity and ramped wall temperature accounting heat radiation and injection/consumption

**DOI:** 10.1038/s41598-020-74739-w

**Published:** 2020-10-20

**Authors:** Talha Anwar, Poom Kumam, Wiboonsak Watthayu

**Affiliations:** 1 Department of Mathematics, Faculty of Science, King Mongkut’s University of Technology Thonburi (KMUTT), 126 Pracha-Uthit Road, Bang Mod, Thung Khru, Bangkok 10140 Thailand; 2 KMUTTFixed Point Research Laboratory, Room SCL 802 Fixed Point Laboratory, Science Laboratory Building, Department of Mathematics, Faculty of Science, King Mongkut’s University of Technology Thonburi (KMUTT), 126 Pracha-Uthit Road, Bang Mod, Thung Khru, Bangkok 10140 Thailand; 3 Center of Excellence in Theoretical and Computational Science (TaCS-CoE), Science Laboratory Building, King Mongkut’s University of Technology Thonburi (KMUTT), 126 Pracha-Uthit Road, Bang Mod, Thung Khru, Bangkok 10140 Thailand; 4 Department of Medical Research, China Medical University Hospital, China Medical University, Taichung, 40402 Taiwan

**Keywords:** Engineering, Mathematics and computing, Nanoscience and technology, Physics

## Abstract

This article investigates the influence of ramped wall velocity and ramped wall temperature on time dependent, magnetohydrodynamic (MHD) natural convection flow of some nanofluids close to an infinitely long vertical plate nested in porous medium. Combination of water as base fluid and three types of nanoparticles named as copper, titanium dioxide and aluminum oxide is taken into account. Impacts of non linear thermal radiation flux and heat injection/consumption are also evaluated. The solutions of principal equations of mass and heat transfer are computed in close form by applying Laplace transform. The physical features of connected parameters are discussed and elucidated with the assistance of graphs. The expressions for Nusselt number and skin friction are also calculated and control of pertinent parameters on both phenomenons is presented in tables. A comparative study is performed for ramped wall and isothermal wall to evaluate the application extent of both boundary conditions.

## Introduction

In modern times, nanotechnology is attracting researchers and scientists for its practical utilities in engineering and industrial sciences. Contemporaneously, nanoliquids are involved in heating and cooling processes such as calming down the nuclear reactors, minimizing the temperature of radiators in vehicles, handling the heat generation in computer processes and controlling thermal flows through heat valves. In pharmaceutical industry, diagnoses and treatment of cancer is based on nanoliquid operators which comprise of different radiations. These noteworthy physical attributes of nanofluids and their implications are fascinating scientists and researchers. The term nanofluid is referred to addition of some solid nanoparticles in regular fluid, sometimes known as base fluid. This idea was first introduced by Choi^1^. Nanoparticles have the tendency to elevate the thermal conductivity of usual fluids such as water, ethylene glycol and mineral oils. The formation of nanoparticles comprises of carbides, metals and carbon nanotubes. Some practical utilities of nanoparticles these days are such as, vehicles have more lighter weight bumpers, cars have sunscreens which provide resistant to radiations, stronger synthetic bones, several sports balls are more durable and clothes are stain repellent. In addition, in the modern era of nanotechnology, where each object is getting enrich in features and reducing in size, nano-catalysts have significant applications in numerous process like water purification, drugs delivery, bio diesel production, solid rocket propellants and formation of carbon nanotubes^2^. As reported by Masuda et al.^3^, nanofluid has higher thermal conductivity due to addition of nanoparticles, but certainly it has different structure depending upon the size and shape of nanoparticles. Das et al.^4^ presented two to four times enhancement in thermal conductivity of $\mathrm {Al_2O_3}$-water and $\mathrm {TiO_2}$-water nanofluids for a small temperature range of 21–51 °C.

The study of mass and thermal flows of incompressible, viscous nanofluids is highly significant because of essential applications of such flows in engineering, chemistry and physics. Imposition of external magnetic field and placement of cavities filled with fluid and porous medium affect the flow of electrically insulated fluid in bearings, pumps, MHD motors, and generators. Such cavities can be portioned as horizontal cavities^5,6^ and vertical cavities^7,8^. From the variety of purposeful applications of these cavities in industrial and environmental sciences, a few are named as thermal insulation, cooling of nuclear fuel, solar collectors and solidification. Hamad et al.^9^ examined the characteristics of naturally convective flow of nanofluid over a semi-infinite vertical plate in existence of external magnetic field. Das and Jana^10^ investigated the influence of magnetic field on nanofluid flow over an infinite vertical plate. An exact analysis of mass and heat transfer for MHD slip flow of nanofluids is provided by Turkyilmazoglu^11^. Sheikholeslami and Ganji^12^ numerically studied the flow of nanofluid over a permeable surface in rotating system. Hussanan et al.^13^ examined unsteady flow of some nanofluids over an accelerating wall nested in porous media in presence of magnetic field. Problems associated to modeling of heat and mass transfer flows in porous material are discussed by Amhalhel et al.^14^. The impact of using porous moving wall for forced MHD laminar flow corresponding to convective boundary conditions was investigated by AbdEl-Gaied et al.^15^. Wang et al.^16^ theoretically analyzed the formation of vortex in magnetized superfluids by constructing the exact solutions through similarity transformation. Turkyilmazoglu^17^ derived analytical solutions for momentum and energy transfer of MHD natural convective nanofluids motion over an instinctive upright wall. Mass transfer in porous stretching surface generating nonlinear MHD flow was reported by Singh et al.^18^.

The investigation of thermal flow features of transient, MHD natural convective flow of viscous fluids with insertion of solid nanoparticles is extremely valuable due to practicability of such fluids in heat transfer instruments. Nanofluids have wide range of applications in numerous engineering process like advanced nuclear power plants and space aircraft due to convective heat transfer rates and higher thermal conductivity^19^. The other prime factors which can effectively control the rate of heat transfer are thermal radiation and heat injection/consumption. These factors have variety of practical utilities in food processing, ventilation, heat treatment and air conditioning^20^. Welding mechanics and thermal engineering deals with addition of heat sources or sinks to free and forced convective MHD flows to optimize the efficiency of cooling and heating processes^21,22^. Heat absorption/generation effects for MHD natural convective nanofluid flow over a vertical plate were reported by Chamkha and Aly^23^. Turkyilmazoglu and Pop^24^ conducted a theoretical study to analyze the radiation effects on MHD natural convection flow of nanofluids passing a vertical stretching sheet. Sheikholeslami et al.^25^ operated two phase model to analyze the impacts of heat radiation flux on heat transfer and MHD flow of nanofluids. Li et al.^26^ proved the global stability of nonlinear equations based ferromagnetic type solitons with the assistance of energy comparison. Influence of heat injection/consumption on nanofluid stagnation point flow was discussed by Soomro et al.^27^. Hamad and Pop^28^ studied and discussed the time dependent MHD natural convective nanofluid motion over a permeable flat vertical wall in a revolving frame of reference with constant heat generation. Reddy^29^ investigated the impacts of thermal radiation and heat generation for a micro-polar fluid flow over a stretching surface. Khan et al.^30^ inspected heat transfer phenomenon for MHD flow of Casson type nanofluid in presence of heat generation/consumption and thermal radiation. Some identical investigations can be studied in^31–35^.

However, all these efforts were made for uniform boundary conditions only, though ramped boundary conditions have enormous significant applications. According to authors’ knowledge there is no single article in literature which deals with simultaneous application of ramped velocity and ramped temperature at wall for unsteady natural convective MHD nanofluid mass and heat transfer. The principal reason behind this shortfall is that resulting mathematical relations are extremely intricate and handling them analytically is sometimes troublesome. The idea of operating ramped wall velocity and ramped wall temperature at the same time was first initiated by Ahmed and Dutta^36^ for unsteady flow and mass transfer of Newtonian fluid passing an impulsively moving vertical plate. Operating ramped wall temperature and ramped wall velocity is highly significant in various subdivisions of present-day technology and science. For instance, ramped velocity is useful in evaluating the functioning of heart and blood vessels. Diagnoses of cardiovascular deceases, determining treatment and establishing prognosis involve treadmill testing and Ergometry, which operate on the basis of ramped velocity^37^. Bruce^38^ reported ramped velocity based analysis which provides the functional tolerance and exercise limitations of cardiac patients. Furthermore, ramped exercise protocols for clinical exercise testing were investigated by Myers and Bellin^39^.

The credit of considering non-uniform (ramped or time-dependent) temperature conditions may be awarded to pioneer studies of Malhotra et al.^40^, Schetz^41^ and Hayday^42^. There are numerous methods available in chemical industry for the management of hazard material through thermal screening. To name a few only, e.g., Insulated Exotherm Test (IET), Differential Scanning Calorimeter (DSC), Thermal Screening Units (TSU), Differential Thermal Analysis, The Carius tube apparatus. With existence of these methods, ramped heating is an efficient technique to handle the anticipation of temperature rise under adiabatic conditions. Another significant practicability of time dependent temperature condition was highlighted by Kundu^43^. He reported that the purpose of destroying cancerous cells can be achieved by thermal therapy since time dependent temperature condition allows to reduce the side effects of this therapy to almost non-existence. Moreover, Kundu^43^ suggested five dissimilar kinds of Fourier and non-Fourier heating based boundary conditions to optimize the effectiveness of the cancer treatment. Keolyar et al.^44^ examined unsteady radiative MHD flow of a nanofluid passing a flat plate with controlled temperature condition. Impact of ramped wall temperature boundary condition on convective viscous fluid flow was evaluated by Chandran et al.^45^. Seth et al.^46^ further elaborated this analysis of ramed wall temperature by considering the plate nested in porous medium. Narahari et al.^47^ used ramped wall temperature at boundary to discuss the influence of mass transfer on viscous convective fluid flow passing an infinite vertical plate. Seth et al.^48–50^ gave attention to practical features of heat and mass transfer under different physical phenomenons like Hall current, chemical reaction and Darcy’s law for impulsive/accelerating motion of plate subjected to ramped temerature at the boundary. Zin et al.^51^ provided a comprehensive analysis of considering ramped temperature condition for transient MHD natural convection flow of Jaffery fluid passing over an upright wall. Maqbool et al.^52^ further extended this study by adding the ramped wall velocity condition at wall and porosity of the medium.

The primary goal of this investigation is to analyze the influence of simultaneous application of ramped wall temperature and ramped wall velocity on unsteady, natural convective flow of water based nanofluids passing an infinite vertical plate nested in porous medium. Along the direction perpendicular to the plate, a uniform magnetic field is imposed in existence of thermal radiative flux and heat injection/consumption. The nanofluids of three types containing water as base fluid along with nanoparticles of Copper (Cu), Titanium dioxide ($\mathrm {TiO_2}$) and Aluminum oxide ($\mathrm {Al_2O_3}$) are chosen in this work. The nonlinear heat radiation flux is linearized with the aid of Taylor series. Employing ramped boundary conditions simultaneously results in intricate mathematical expressions which involve branch points and poles. Consequently, evaluation of inverse Laplace transformation becomes extremely burdensome. However in present work, exact solutions of momentum and energy equations are calculated by implementing Laplace transform and provided in close form. The dependence of velocity and thermal profiles on several connected parameters is interpreted with the assistance of graphs. The relations for Nusselt number and skin friction are computed and analyzed.

## Mathematical modeling

The principal governing equations of an incompressible free convective MHD flow and energy transfer in existence of nonlinear thermal radiative flux and heat injection/consumption of a fluid past an infinitely long vertical plate nested in a porous medium subject to Boussinesq’s approximation are given as^36,53,54^.

1 $$\begin{aligned}&\nabla . \mathbf {V}=0, \end{aligned}$$

2 $$\begin{aligned}&\rho \left[ \frac{\partial {\mathbf {V}}}{\partial t}+\mathbf {(V.\nabla )} {\mathbf {V}} \right] = {\mathbf {r}}+\mu \nabla ^2 {\mathbf {V}}+\mathbf {J \times B}+\rho {\mathbf {g}} \beta (\Theta -\Theta _\infty ), \end{aligned}$$

3 $$\begin{aligned}&\rho C_p \left[ \frac{\partial \Theta }{\partial t}+\mathbf {(V.\nabla )} \Theta \right] =K_f \mathbf {\nabla ^2}\Theta +\gamma _1-\frac{\partial Q_r}{\partial n}-Q_0(\Theta -\Theta _\infty ). \end{aligned}$$

Consider unsteady natural convective fluid flow and energy transfer of a nanofluid past an infinitely long vertical plate nested in porous medium. Initially, both the plate and fluid are static at same temperature $\Theta _\infty$. At time $t>0$, the plate starts an impulsive motion with velocity $U_0 \frac{t}{t_0}$ and temperature of vertical plate is raised to $\Theta _\infty +(\Theta _w-\Theta _\infty )\frac{t}{t_0}$ for $0<t \le t_0$. Later on, a uniform velocity $U_0$ and constant temperature $\Theta _w$ is maintained for $t>t_0$. Assuming that flow is one dimensional and unidirectional, *x-*axis is considered in direction parallel to the vertical plate and *y*-axis is chosen perpendicular to the plate. The plate is considered to be situated at $y=0$ and nanofluid flow is restricted to $y>0$. Furthermore, assumptions made to idealize the considered model are mentioned as

- The nanofluid is comprised of base fluid water and nanoparticles named as Cu, $\mathrm {TiO_2}$ and $\mathrm {Al_2O_3}$.
- Thermal equilibrium is maintained between base fluid and nanoparticles.
- Temperature buoyancy force in velocity equation is function of density.
- It is assumed that thermal radiative flux ($Q_r$) has sufficiently small physical effect in direction parallel to the plate such that it can be neglected.
- In energy equation, viscous dissipation term is neglected.
- The resulting magnetic field because of nanofluid flow is neglected as compared to imposed magnetic field.
- Polarization effect of nanofluid is neglected in such a way that no external electric field is employed.
- It is considered that nanoparticles have uniform shape and size.

**Figure 1:**
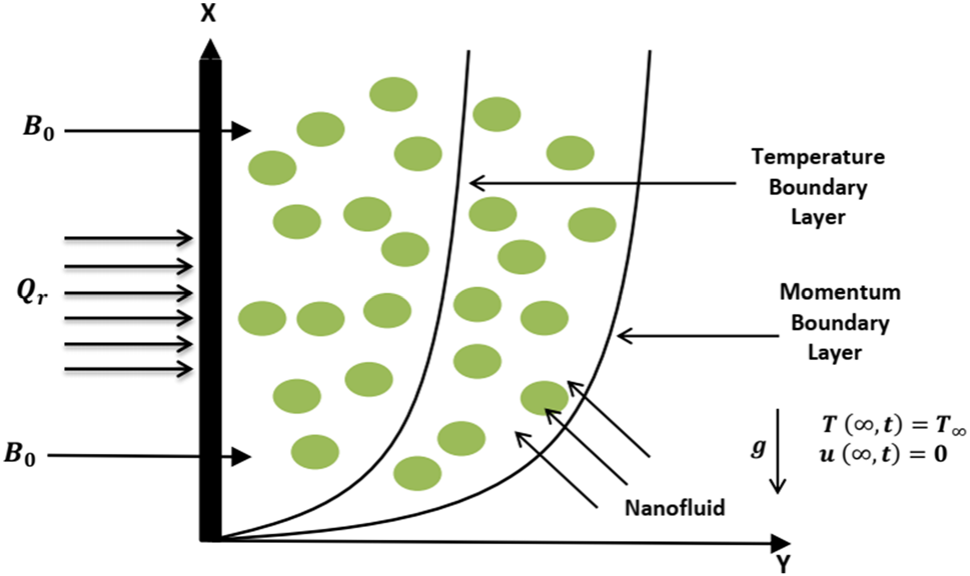
Geometry of the considered model.

Since one dimensional and unidirectional flow is considered and it is assumed that vertical plate has infinite length, therefore only variation in *t* and *y* affect the temperature and velocity of nanofluid. The geometrical interpretation of physical model is provided in Fig. 1.

The Darcy’s law encounters the Newtonian fluid in following manner

4 $$\begin{aligned} {\mathbf {r}}=-\frac{\mu \gamma _2}{k^*}u. \end{aligned}$$

The Maxwell’s equations to deal with magnetic field are given as

5 $$\begin{aligned} \mathrm {div {\mathbf {B}}}=0, \quad \mathrm {curl} {\mathbf {B}}=\mu _m {\mathbf {J}}, \quad \mathrm {Curl} {\mathbf {E}}=-\frac{\partial {\mathbf {B}}}{\partial t}, \end{aligned}$$

and use of Ohm’s law further leads to

6 $$\begin{aligned} \mathbf {J \times B}=-(\sigma B^2_0 u,0,0). \end{aligned}$$

In the light of Eqs. (4)–(6) and all aforementioned assumptions, Eqs. (2) and (3) for a nanofluid can be expressed as

7 $$\begin{aligned} \rho _{nf} \frac{\partial u}{\partial t}=&\mu _{nf} \frac{\partial ^2 u }{\partial y^2}-\frac{\mu _{nf} \gamma _2}{k^*}u+g(\rho \beta )_{nf} (\Theta -\Theta _\infty )-\sigma _{nf} B^2_0 u, \end{aligned}$$

8 $$\begin{aligned} (\rho C_p)_{nf}\frac{\partial \Theta }{\partial t}=&K_{nf} \frac{\partial ^2 \Theta }{\partial y^2}-\frac{\partial Q_r}{\partial y}-Q_0 (\Theta -\Theta _\infty ). \qquad \end{aligned}$$

The initial and boundary conditions corresponding to momentum and energy equations are respectively stated as

9 $$\begin{aligned}&u(y,0)=0, \quad \Theta (y,0)=\Theta _\infty \quad \text {for} \quad y \ge 0, \end{aligned}$$

10 $$\begin{aligned}&u(0,t)=\left\{ \begin{array}{ll} U_0\frac{t}{t_0} \quad 0 < t \le t_0 \\ U_0 \qquad t>t_0, \end{array}\right. \end{aligned}$$

11 $$\begin{aligned}&\Theta (0,t)=\left\{ \begin{array}{ll} \Theta _\infty +(\Theta _w-\Theta _\infty )\frac{t}{t_0} \quad 0 < t \le t_0 \\ \Theta _w \qquad \qquad \qquad \qquad t>t_0, \end{array}\right. \end{aligned}$$

12 $$\begin{aligned}&u(y,t) \rightarrow 0, \quad \Theta (y,t) \rightarrow \Theta _\infty , \nonumber \\&\text {when} \quad y \rightarrow \infty \quad \text {for} \quad t>0. \end{aligned}$$

The expressions for dynamic viscosity $\mu _{nf}$, heat capacitance $(\rho C_p)_{nf}$, coefficient of thermal expansion $(\rho \beta )_{nf}$, density $\rho _{nf}$ and electrical conductivity $\sigma _{nf}$ of nanofluid are respectively calculated as^55^

13 $$\begin{aligned} \mu _{nf}= \, &\frac{\mu _w}{(1-\phi )^{2.5}}, \quad (\rho C_p)_{nf}=(\rho C_p)_w \left[ 1-\phi +\phi \frac{(\rho C_p)_{np}}{(\rho C_p)_w} \right] , \nonumber \\ (\rho \beta )_{nf}= \, &(\rho \beta )_w \left[ 1-\phi +\phi \frac{(\rho \beta )_{np}}{(\rho \beta )_w} \right] , \quad \rho _{nf}=\rho _w \left[ 1-\phi +\phi \frac{\rho _{np}}{\rho _w} \right] , \nonumber \\&\sigma _{nf}=\sigma _w \left[ 1+ \frac{3 \phi (\sigma -1)}{(\sigma +2)-\phi (\sigma -1)} \right] , \qquad \sigma =\frac{\sigma _{np}}{\sigma _w}. \end{aligned}$$

Hamilton and Crosser model is applied to effectively anticipate the the thermal conductivity of nanoparticles^24,56^.

14 $$\begin{aligned} \frac{K_{nf}}{K_w}=\frac{K_{np}+2K_w-2(K_w-K_{np})\phi }{K_{np}+2K_w+(K_w-K_{np})\phi }. \end{aligned}$$

In Eqs. (7), (8), (13) and (14), the subscripts *w*, *np* and *nf* are associated to the properties of base fluid water, nanoparticles and nanofluid respectively. Moreover it is significant to mention that relations (13) are confined to spherical shape nanoparticles. The assumption of optically thick fluid and Rosseland approximation^57,58^ leads to the following expression of radiation heat flux

15 $$\begin{aligned} Q_r=-\frac{4 \sigma _r}{3 k_r}\frac{\partial \Theta ^4}{\partial y}. \end{aligned}$$

From above expression, it is clear that heat radiation flux is non-linear function of temperature. However, it can be linearized with the assumption that during nanofluid flow, temperature differences are sufficiently small. Expansion of Taylor series of $\Theta ^4$ around uniform ambient temperature $\Theta _\infty$ and elimination of higher order terms on the basis of previous assumption leads to the following linear relation

16 $$\begin{aligned} \Theta ^4 \approx 4 \Theta \Theta^3 _\infty -3 \Theta ^4_\infty . \end{aligned}$$

In the light of Eqs. (15) and (16), Eq. (8) turns out as

17 $$\begin{aligned} (\rho C_p)_{nf}\frac{\partial \Theta }{\partial t}=\left( K_{nf}+\frac{16 \sigma _r \Theta ^3_\infty }{3 k_r} \right) \frac{\partial ^2 \Theta }{\partial y^2}-Q_0 (\Theta -\Theta _\infty ). \end{aligned}$$

Some non-dimensional variables are introduced as follows

18 $$\begin{aligned} u^*=\frac{u}{U_0}, \quad \xi =\frac{y U_0}{\nu _w}, \quad \tau =\frac{t U^2_0}{\nu _w}, \quad \theta =\frac{\Theta -\Theta _\infty }{\Theta _w-\Theta _\infty }. \end{aligned}$$

Employing above dimensionless terms together with Eqs. (13) and (14) in Eqs. (7) and (17) and dropping the * notation on *u* for the sake of brevity, we acquire the following dimensionless coupled system of partial differential equations

19 $$\begin{aligned} \varphi _1 \frac{\partial u}{\partial \tau }=\,&\varphi _4 \frac{\partial ^2 u}{\partial \xi ^2}+\varphi _2 Gr \theta - \varphi _3 M u- \varphi _4\frac{u}{K}, \end{aligned}$$

20 $$\begin{aligned} \frac{\partial \theta }{\partial \tau }=&\left( \frac{\varphi _5+Nr}{\varphi _6 Pr}\right) \frac{\partial ^2 \theta }{\partial \xi ^2}-\frac{Q}{\varphi _6}\theta , \quad \end{aligned}$$

where

21 $$\begin{aligned} \varphi _1 \, &=\left[ 1-\phi +\phi \left( \frac{\rho _{np}}{\rho _w}\right) \right] , \quad \varphi _2=\left[ 1-\phi +\phi \frac{(\rho \beta )_{np}}{(\rho \beta )_w} \right] , \nonumber \\ \varphi _3 \, &=\left[ 1+ \frac{3 \phi (\sigma -1)}{(\sigma +2)-\phi (\sigma -1)} \right] , \quad \varphi _4=\frac{1}{(1-\phi )^{2.5}}, \nonumber \\ \varphi _5 \, &=\frac{K_{np}+2K_w-2(K_w-K_{np})\phi }{K_{np}+2K_w+(K_w-K_{np})\phi }, \quad \varphi _6=\left[ 1-\phi +\phi \frac{(\rho C_p)_{np}}{(\rho C_p)_w} \right] , \nonumber \\ Gr \, &=\frac{g (\Theta _w-\Theta _\infty )(\nu \beta )_w}{U_0^3}, \quad Pr=\left( \frac{\mu C_p}{K}\right) _w, \quad \frac{1}{K}=\frac{\gamma _2 \nu _w^2}{U_0^2k^*}, \nonumber \\ M&=\frac{B_0^2}{U_0^2}\left( \frac{\sigma \nu }{\rho }\right) _w, \quad Nr=\frac{16 \sigma _r \Theta _\infty ^3}{3 k_r K_w}, \quad Q=\frac{Q_0}{U_0^2} \left( \frac{\nu }{\rho C_p} \right) _w. \end{aligned}$$

The dimensionless form of initial and boundary conditions is determined as

22 $$\begin{aligned}&u(\xi ,0)=0, \quad \theta (\xi ,0)=0 \quad \text {for} \quad \xi \ge 0, \end{aligned}$$

23 $$\begin{aligned}&u(0,\tau )=\theta (0,\tau )=\left\{ \begin{array}{ll} \tau \quad 0 < \tau \le 1 \\ 1 \qquad \tau >1, \end{array}\right. \end{aligned}$$

24 $$\begin{aligned}&u(\xi ,\tau ) \rightarrow 0, \quad \theta (\xi ,\tau ) \rightarrow 0 \quad \text {as} \quad \xi \rightarrow \infty \quad \text {for} \quad \tau >0. \end{aligned}$$

## Analytical solutions

Laplace transformation^59^ is an efficient tool to derive the solution of present problem, since the numerous traditional techniques such as separation of variables, perturbation method and Homotopy analysis method fail to overcome the complexity of time controlled boundary conditions. Formulation of integral form of Laplace transform pair to evaluate the results of considered model is proposed as

25 $$\begin{aligned} {\bar{R}}(\xi ,p)=\int \limits _{0}^{\infty } e^{-p \tau }R(\xi ,\tau )d\tau ={\mathcal {L}}[R](\tau ). \end{aligned}$$

In current problem, $R\in \{\theta , u\}$. The condition $Re(p)>\gamma _0$ guarantees the convergence of integral in Eq. (25), where $\gamma _o$ is an arbitrary real constant and $p=\Psi +i\Omega$, with $i=\sqrt{-1}$. The integral form of inverse Laplace transformation to obtain the solutions in real time domain is given as

26 $$\begin{aligned} R(\xi ,\tau )=\frac{1}{2\pi i}\int \limits _{BR}e^{p \tau } {\bar{R}}(\xi ,p)dp={\mathcal {L}}^{-1}[{\bar{R}}](p), \end{aligned}$$

### Temperature field

Applying the definition of Laplace transform provided in Eq. (25) on Eqs. (20), (23)_2_, (24)_2_ and plugging Eq. (22) yields

27 $$\begin{aligned}&\frac{d^2 {\bar{\theta }}}{d \xi ^2}- (p \alpha + \lambda ){\bar{\theta }}=0, \end{aligned}$$

28 $$\begin{aligned}&{\bar{\theta }}(0,p)=\frac{1-e^{-p}}{p^2}, \quad {\bar{\theta }}(\xi ,p) \rightarrow 0 \quad \text {as} \quad \xi \rightarrow \infty , \end{aligned}$$

where

29 $$\begin{aligned} \alpha =\frac{\varphi _6 Pr}{\varphi _5+Nr}, \quad \lambda = \frac{Q Pr}{\varphi _5+Nr}. \end{aligned}$$

The solution of ordinary differential Eq. (27) corresponding to boundary conditions (28) is derived as

30 $$\begin{aligned} {\bar{\theta }}(\xi ,p)=\left( \frac{1-e^{-p}}{p^2}\right) e^{-\sqrt{p \alpha +\lambda }\xi }. \end{aligned}$$

Implementing inverse Laplace transformation provided in Eq. (26) on Eq. (30) emits

31 $$\begin{aligned} \theta (\xi ,\tau )=\psi _1-{\hat{\psi }}_1 \times G(\tau -1), \end{aligned}$$

where

32 $$\begin{aligned} \psi _1 \left( \frac{1}{\alpha }, \frac{1}{\lambda }, \xi , \tau \right) \, &= \, \frac{1}{2}\left[ \left( \tau +\frac{\alpha \xi }{2} \sqrt{\frac{1}{\lambda }} \right) e^{\xi \sqrt{\lambda }} {\text {erfc}} \left( \frac{\xi }{2} \sqrt{\frac{\alpha }{\tau }}+\sqrt{\frac{\lambda \tau }{\alpha } }\right) \right. \nonumber \\ +&\left. \left( \tau -\frac{\alpha \xi }{2} \sqrt{\frac{1}{\lambda }} \right) e^{-\xi \sqrt{\lambda }} {\text {erfc}} \left( \frac{\xi }{2} \sqrt{\frac{\alpha }{\tau }}-\sqrt{\frac{\lambda \tau }{\alpha } }\right) \right] , \nonumber \\ {\hat{\psi }}_1\left( \frac{1}{\alpha }, \frac{1}{\lambda }, \xi , \tau -1 \right)&= \, \frac{1}{2}\left[ \left( \tau -1+\frac{\alpha \xi }{2} \sqrt{\frac{1}{\lambda }} \right) e^{\xi \sqrt{\lambda }} {\text {erfc}} \left( \frac{\xi }{2} \sqrt{\frac{\alpha }{\tau -1}}+\sqrt{\frac{\lambda (\tau -1)}{\alpha } }\right) \right. \nonumber \\ +&\left. \left( \tau -1-\frac{\alpha \xi }{2} \sqrt{\frac{1}{\lambda }} \right) e^{-\xi \sqrt{\lambda }} {\text {erfc}} \left( \frac{\xi }{2} \sqrt{\frac{\alpha }{\tau -1}}-\sqrt{\frac{\lambda (\tau -1)}{\alpha } }\right) \right] , \end{aligned}$$

with $G(\tau -1)$ denoting a Heaviside function.

### Velocity field

Employing Laplace transform on Eqs. (19), (23)_1_, (24)_1_ and using Eq. (22) gives

33 $$\begin{aligned}&\frac{d^2 {\bar{u}}}{d \xi ^2}-(p \eta +\omega ){\bar{u}}=- Grm {\bar{\theta }}, \end{aligned}$$

34 $$\begin{aligned}&{\bar{u}}(0,p)=\frac{1-e^{-p}}{p^2}, \quad {\bar{u}}(\xi ,p) \rightarrow 0 \quad \text {as} \quad \xi \rightarrow \infty , \end{aligned}$$

where

35 $$\begin{aligned} \eta =\frac{\varphi _1}{\varphi _4}, \quad \omega =M \frac{\varphi _3}{\varphi _4}+\frac{1}{K}, \quad m=\frac{\varphi _2}{\varphi _4}. \end{aligned}$$

Introducing Eqs. (30) into (33) results in the following form

36 $$\begin{aligned} \frac{d^2 {\bar{u}}}{d \xi ^2}-(p \eta +\omega ){\bar{u}}=- Gr m \left( \frac{1-e^{-p}}{p^2}\right) e^{-\sqrt{p \alpha +\lambda }\xi }. \end{aligned}$$

The solution of ordinary differential Eq. (36) subjected to boundary conditions (34) is computed as

37 $$\begin{aligned} {\bar{u}}(\xi ,\tau )=\left( \frac{1-e^{-p}}{p^2}\right) \left[ e^{-\sqrt{p \eta +\omega } \xi }+\frac{Gr m}{a (p-b )}\left\{ e^{-\sqrt{p \eta +\omega } \xi }-e^{-\sqrt{p \alpha +\lambda }\xi } \right\} \right] , \end{aligned}$$

where

38 $$\begin{aligned} a=\alpha -\eta , \qquad b=\frac{\omega -\lambda }{\alpha -\eta }. \end{aligned}$$

Operating inverse Laplace transform on Eq. (37) results as

39 $$\begin{aligned} u(\xi ,\tau )=\psi _2-{\hat{\psi }}_2 \times G(\tau -1)+\frac{ Gr m }{a} [\psi _3 - {\hat{\psi }}_3+\psi _4 - {\hat{\psi }}_4], \end{aligned}$$

where

40 $$\begin{aligned} \psi _2 \left( \frac{1}{\eta },\frac{1}{\omega },\xi ,\tau \right) = \, &\frac{1}{2}\left[ \left( \tau +\frac{\xi \eta }{2} \sqrt{\frac{1}{\omega }} \right) e^{\xi \sqrt{\omega }} {\text {erfc}} \left( \frac{\xi }{2} \sqrt{\frac{\eta }{\tau }}+\sqrt{\frac{\omega \tau }{\eta } }\right) \right. \nonumber \\&\left. \left( \tau -\frac{\xi \eta }{2} \sqrt{\frac{1}{\omega }} \right) e^{-\xi \sqrt{\omega }} {\text {erfc}} \left( \frac{\xi }{2} \sqrt{\frac{\eta }{\tau }}-\sqrt{\frac{\omega \tau }{\eta } }\right) \right] , \nonumber \\ \hat{\psi _2} \left( \frac{1}{\eta },\frac{1}{\omega },\xi ,\tau -1 \right) = \, &\frac{1}{2}\left[ \left( \tau -1+\frac{\xi \eta }{2} \sqrt{\frac{1}{\omega }} \right) e^{\xi \sqrt{\omega }} {\text {erfc}} \left( \frac{\xi }{2} \sqrt{\frac{\eta }{\tau -1}}+\sqrt{\frac{\omega (\tau -1)}{\eta } }\right) \right. \nonumber \\&\left. \left( \tau -1-\frac{\xi \eta }{2} \sqrt{\frac{1}{\omega }} \right) e^{-\xi \sqrt{\omega }} {\text {erfc}} \left( \frac{\xi }{2} \sqrt{\frac{\eta }{\tau -1}}-\sqrt{\frac{\omega (\tau -1)}{\eta } }\right) \right] , \nonumber \\ \psi _3= \, &\frac{1}{2b^2 }e^{b \tau }(E_1-E_2), \quad {\hat{\psi }}_3=\frac{1}{2b^2 }e^{b (\tau -1)}({\hat{E}}_1-{\hat{E}}_2)\times G(\tau -1), \nonumber \\ \psi _4= \, &\frac{1}{2b }(F_1-F_2), \qquad {\hat{\psi }}_4=\frac{1}{2b}({\hat{F}}_1-{\hat{F}}_2)\times G(\tau -1), \nonumber \\ E_1\left( \frac{1}{\eta }, \frac{1}{\omega },b,\xi ,\tau \right) = \, &e^{\xi \sqrt{\eta b+\omega }} {\text {erfc}} \left( \frac{\xi }{2}\sqrt{\frac{\eta }{\tau }}+\sqrt{(b+\eta \omega )\tau }\right) \nonumber \\&+ e^{-\xi \sqrt{\eta b+\omega }} {\text {erfc}} \left( \frac{\xi }{2}\sqrt{\frac{\eta }{\tau }}-\sqrt{(b+\eta \omega )\tau }\right) , \nonumber \\ E_2\left( \frac{1}{\alpha }, \frac{1}{\lambda },b,\xi ,\tau \right) = \, &e^{\xi \sqrt{\alpha b+\lambda }} {\text {erfc}} \left( \frac{\xi }{2}\sqrt{\frac{\alpha }{\tau }}+\sqrt{(b+\alpha \lambda )\tau }\right) \nonumber \\&+ e^{-\xi \sqrt{\alpha b+\lambda }} {\text {erfc}} \left( \frac{\xi }{2}\sqrt{\frac{\alpha }{\tau }}-\sqrt{(b+\alpha \lambda )\tau }\right) , \nonumber \\ {\hat{E}}_1\left( \frac{1}{\eta }, \frac{1}{\omega },b,\xi ,\tau -1 \right) = \, &e^{\xi \sqrt{\eta b+\omega }} {\text {erfc}} \left( \frac{\xi }{2}\sqrt{\frac{\eta }{\tau -1}}+\sqrt{(b+\eta \omega )(\tau -1)}\right) \nonumber \\&+ e^{-\xi \sqrt{\eta b+\omega }} {\text {erfc}} \left( \frac{\xi }{2}\sqrt{\frac{\eta }{\tau -1}}-\sqrt{(b+\eta \omega )(\tau -1)}\right) , \nonumber \\ {\hat{E}}_2\left( \frac{1}{\alpha }, \frac{1}{\lambda },b,\xi ,\tau -1\right) = \, &e^{\xi \sqrt{\alpha b+\lambda }} {\text {erfc}} \left( \frac{\xi }{2}\sqrt{\frac{\alpha }{\tau -1}}+\sqrt{(b+\alpha \lambda )(\tau -1)}\right) \nonumber \\&+ e^{-\xi \sqrt{\alpha b+\lambda }} {\text {erfc}} \left( \frac{\xi }{2}\sqrt{\frac{\alpha }{\tau -1}}-\sqrt{(b+\alpha \lambda )(\tau -1)}\right) , \nonumber \\ F_1\left( \frac{1}{\eta }, \frac{1}{\omega },b,\xi ,\tau \right) = \, &\left( \tau +\frac{\xi \eta }{2} \sqrt{\frac{1}{\omega }}+\frac{1}{b}\right) e^{\xi \sqrt{\omega }}{\text {erfc}} \left( \frac{\xi }{2}\sqrt{\frac{\eta }{\tau }}+\sqrt{\frac{\omega \tau }{\eta }} \right) \nonumber \\&+\left( \tau -\frac{\xi \eta }{2} \sqrt{\frac{1}{\omega }}+\frac{1}{b}\right) e^{-\xi \sqrt{\omega }}{\text {erfc}} \left( \frac{\xi }{2}\sqrt{\frac{\eta }{\tau }}-\sqrt{\frac{\omega \tau }{\eta }} \right) , \nonumber \\ F_2\left( \frac{1}{\alpha }, \frac{1}{\lambda },b,\xi ,\tau \right) = \, &\left( \tau +\frac{\xi \alpha }{2} \sqrt{\frac{1}{\lambda }}+\frac{1}{b}\right) e^{\xi \sqrt{\lambda }}{\text {erfc}} \left( \frac{\xi }{2}\sqrt{\frac{\alpha }{\tau }}+\sqrt{\frac{\lambda \tau }{\alpha }} \right) \nonumber \\&+\left( \tau -\frac{\xi \alpha }{2} \sqrt{\frac{1}{\lambda }}+\frac{1}{b}\right) e^{-\xi \sqrt{\lambda }}{\text {erfc}} \left( \frac{\xi }{2}\sqrt{\frac{\alpha }{\tau }}-\sqrt{\frac{\lambda \tau }{\alpha }} \right) , \nonumber \\ {\hat{F}}_1\left( \frac{1}{\eta }, \frac{1}{\omega },b,\xi ,\tau -1 \right) = \, &\left( \tau -1+\frac{\xi \eta }{2} \sqrt{\frac{1}{\omega }}+\frac{1}{b}\right) e^{\xi \sqrt{\omega }}{\text {erfc}} \left( \frac{\xi }{2}\sqrt{\frac{\eta }{\tau -1}}+\sqrt{\frac{\omega (\tau -1)}{\eta }} \right) \nonumber \\&+ \left( \tau -1-\frac{\xi \eta }{2} \sqrt{\frac{1}{\omega }}+\frac{1}{b}\right) e^{-\xi \sqrt{\omega }}{\text {erfc}} \left( \frac{\xi }{2}\sqrt{\frac{\eta }{\tau -1}}-\sqrt{\frac{\omega (\tau -1)}{\eta }} \right) , \nonumber \\ {\hat{F}}_2\left( \frac{1}{\alpha }, \frac{1}{\lambda },b,\xi ,\tau -1 \right) = \, &\left( \tau -1+\frac{\xi \alpha }{2} \sqrt{\frac{1}{\lambda }}+\frac{1}{b}\right) e^{\xi \sqrt{\lambda }}{\text {erfc}} \left( \frac{\xi }{2}\sqrt{\frac{\alpha }{\tau -1}}+\sqrt{\frac{\lambda (\tau -1)}{\alpha }} \right) \nonumber \\&+\left( \tau -1-\frac{\xi \alpha }{2} \sqrt{\frac{1}{\lambda }}+\frac{1}{b}\right) e^{-\xi \sqrt{\lambda }}{\text {erfc}} \left( \frac{\xi }{2}\sqrt{\frac{\alpha }{\tau -1}}-\sqrt{\frac{\lambda (\tau -1)}{\alpha }} \right) . \end{aligned}$$

### Nusselt number

The expression for rate of heat transfer (or Nusselt number) at wall is derived as

41 $$\begin{aligned} \text {Nu}=-\varphi _5 \frac{\partial \theta }{\partial \xi }\Big |_{\xi =0}, \end{aligned}$$

where

42 $$\begin{aligned} \frac{\partial \theta }{\partial \xi }\Big |_{\xi =0}= \frac{\partial \psi _1}{\partial \xi }\Big |_{\xi =0}-\left( \frac{\partial {\hat{\psi }}_1}{\partial \xi }\Big |_{\xi =0}\right) \times G(\tau -1). \end{aligned}$$

The gradients involved in Eq. (42) are provided in Eqs. (A1) and (A2).

### Skin friction

The skin friction coefficient at wall is computed as

43 $$\begin{aligned} C_f=\varphi _4 \frac{\partial u}{\partial \xi }\Big |_{\xi =0}, \end{aligned}$$

where

44 $$\begin{aligned} \frac{\partial u}{\partial \xi }\Big |_{\xi =0}&=\frac{\partial \psi _2}{\partial \xi }\Big |_{\xi =0}+\left( \frac{\partial {\hat{\psi }}_2}{\partial \xi }\Big |_{\xi =0} \right) \times G(\tau -1)+\frac{ Gr m }{a} \left[ \frac{1}{2 b^2}e^{b \tau }\left( \frac{\partial E_1}{\partial \xi }\Big |_{\xi =0} - \frac{\partial E_2}{\partial \xi }\Big |_{\xi =0} \right) \right. \nonumber \\&\quad -\left. \frac{1}{2 b^2}e^{b (\tau -1)}\left( \frac{\partial {\hat{E}}_1}{\partial \xi }\Big |_{\xi =0} - \frac{\partial {\hat{E}}_2}{\partial \xi }\Big |_{\xi =0} \right) \times G(\tau -1)+\frac{1}{2 b}\left( \frac{\partial F_1}{\partial \xi }\Big |_{\xi =0} - \frac{\partial F_2}{\partial \xi }\Big |_{\xi =0} \right) \right. \nonumber \\&\quad -\left. \frac{1}{2 b}\left( \frac{\partial {\hat{F}}_1}{\partial \xi }\Big |_{\xi =0} - \frac{\partial {\hat{F}}_2}{\partial \xi }\Big |_{\xi =0} \right) \times G(\tau -1) \right] . \end{aligned}$$

The gradients involved in Eq. (44) are presented in Eqs. (A3)–(A5).

## Limiting models

### Case 1

The energy and momentum solutions for isothermal plate temperature and uniform plate velocity take the following form

45 $$\begin{aligned} \theta (\xi ,\tau )&=f_0(\alpha ,\lambda ,\xi ,\tau ), \end{aligned}$$

46 $$\begin{aligned} u(y,t)&= f_1(\eta ,\omega ,\xi ,\tau )+\frac{Grma^*}{b^*} \left[ f_2(\eta ,\omega ,b^*,\xi ,\tau )-f_3(\eta ,\omega ,b^*,\xi ,\tau ) \right. \nonumber \\&\left. -f_1(\eta ,\omega ,\xi ,\tau )+f_0(\alpha ,\lambda ,\xi ,\tau ) \right] , \end{aligned}$$

where

47 $$\begin{aligned} f_0(\alpha ,\lambda ,\xi ,\tau )&=\frac{1}{2}\left[ e^{-\xi \sqrt{\lambda }}{\text {erfc}}\left( \frac{\xi \sqrt{\alpha }}{2\sqrt{\tau }}-\sqrt{\frac{\lambda }{\alpha }\tau }\right) +e^{\xi \sqrt{\lambda }}{\text {erfc}}\left( \frac{\xi \sqrt{\alpha }}{2\sqrt{\tau }}+\sqrt{\frac{\lambda }{\alpha }\tau }\right) \right] , \nonumber \\ f_1(\eta ,\omega ,\xi ,\tau )&=\frac{1}{2}\left[ e^{-\xi \sqrt{\omega }}{\text {erfc}}\left( \frac{\xi \sqrt{\beta ^*}}{2\sqrt{ \tau }}-\sqrt{\frac{\omega }{\eta }\tau }\right) +e^{\xi \sqrt{\omega }}{\text {erfc}}\left( \frac{\xi \sqrt{\beta ^*}}{2\sqrt{ \tau }}+\sqrt{\frac{\omega }{\eta }\tau }\right) \right] , \nonumber \\ f_2(\eta ,\omega ,b^*,\xi ,\tau )&=\frac{e^{b^* \tau }}{2}\left[ e^{-\xi \sqrt{\beta ^*} \sqrt{b^*+\frac{\omega }{\eta }}}{\text {erfc}}\left( \frac{\xi \sqrt{\beta ^*}}{2\sqrt{\tau }}-\sqrt{\left( b^*+\frac{\omega }{\eta }\right) \tau }\right) \right. \nonumber \\ +&\left. e^{\xi \sqrt{\beta ^*} \sqrt{b^*+\frac{\omega }{\eta }}}{\text {erfc}}\left( \frac{\xi \sqrt{\beta ^*}}{2\sqrt{\tau }}+\sqrt{\left( b^*+\frac{\omega }{\eta }\right) \tau }\right) \right] , \nonumber \\ f_3(\alpha ,\lambda ,b^*,\xi ,\tau )&=\frac{e^{b^* \tau }}{2}\left[ e^{-\xi \sqrt{\alpha } \sqrt{b^*+\frac{\lambda }{\alpha }}}{\text {erfc}}\left( \frac{\xi \sqrt{\alpha }}{2\sqrt{\tau }}-\sqrt{\left( b^*+\frac{\lambda }{\alpha }\right) \tau }\right) \right. \nonumber \\ +&\left. e^{\xi \sqrt{\alpha } \sqrt{b+\frac{\lambda }{\alpha }}}{\text {erfc}}\left( \frac{\xi \sqrt{\alpha }}{2\sqrt{\tau }}+\sqrt{\left( b^*+\frac{\lambda }{\alpha }\right) \tau }\right) \right] , \end{aligned}$$

and

48 $$\begin{aligned} a^*=\frac{1}{\alpha \eta -1}, \quad b^*=\frac{\omega -\lambda }{\alpha \eta -1}, \quad \beta ^*=\frac{1}{\eta }. \end{aligned}$$

### Case 2

The cause of authentication of our current results is achieved, when pure viscous fluid $(\phi =0)$ with ramped wall temperature is considered we recover solutions of Seth et al.^46^. In addition, if magnetic parameter $M \rightarrow 0$ and porosity parameter $\frac{1}{K} \rightarrow 0$, we obtain the solutions calculated by Chandran et al.^45^.

## Parametric study

In order to achieve the goal of having comprehensive understanding of physical mechanism of current problem completely, a parametric analysis is performed, and computed solutions are revealed with the assistance of graphs and tables. In this section, solid lines present the solutions of velocity and energy equations with ramped wall velocity and temperature conditions, and similarly dashed lines present solutions under isothermal (constant) wall velocity and temperature conditions. The noteworthy physical attributes of associated parameters such as radiation parameter (Nr), heat injection/consumption parameter (Q), permeability parameter (K), magnetic parameter (M), time ($\tau$), volume fraction of nanoparticles $(\phi )$ and Grashof number (Gr) on dimensionless energy and velocity are investigated and plotted for both ramped and isothermal wall boundary conditions. Extensively, contribution of connected parameters in heat transfer and skin friction is discussed with the aid of tables comprised of numerical computations.

**Figure 2:**
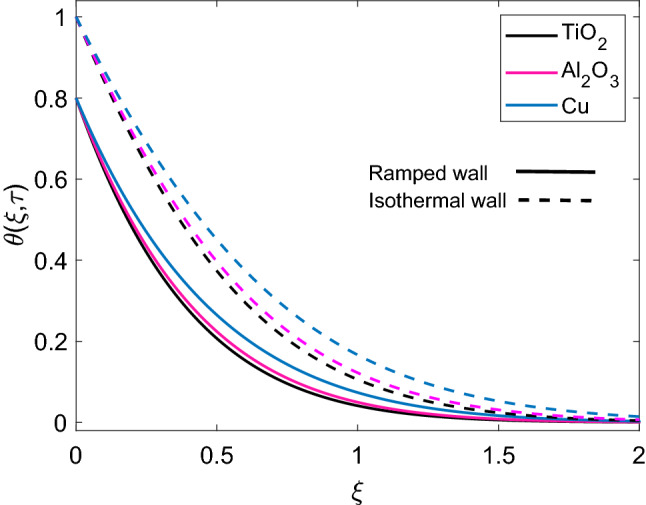
Comparison of temperature profile [Eq. (31)] of different nanofluids when $Nr=0.5$, $\phi =0.1$ and $Q=0.5$.

**Figure 3:**
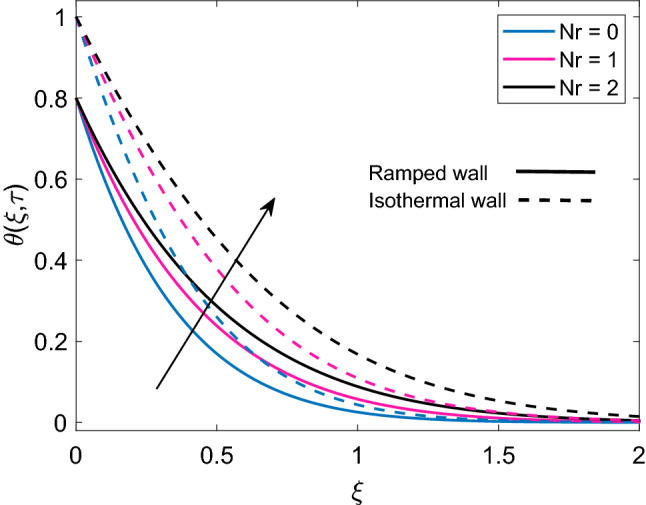
Temperature distribution [Eq. (31)] for various values of *Nr* when $\phi =0.1$ and $Q=0.5$.

Figure 2 illustrates the distribution of dimensionless temperature $(\theta )$, when three different kinds of nanoparticles named as Cu, $\mathrm {Al_2O_3}$ and $\mathrm {TiO_2}$ are suspended in base fluid water. It is witnessed that temperature of Cu-water nanofluid is relatively higher than $\mathrm {Al_2O_3}$-water and $\mathrm {TiO_2}$-water nanofluids. It is obvious because the first nanofluid has much greater thermal conductivity than the later nanofluids. It is also witnessed that since the thermal conductivity of $\mathrm {Al_2O_3}$ and $\mathrm {TiO_2}$ are close enough therefore, the corresponding temperature curves are passing at at very small distance to each other. Furthermore, implementation of ramped wall temperature boundary condition leads to decay the temperature profile. It is noted that Cu-water has more thick temperature boundary layer in contrast to $\mathrm {Al_2O_3}$ and $\mathrm {TiO_2}$. The distribution of dimensionless temperature for ramped wall condition and isothermal wall condition, corresponding to variation of radiation parameter (Nr) is plotted in Figure 3. In both cases, temperature profile follows similar trend, however, it has higher profile for isothermal wall. It is seen that increase in Nr enhances the temperature of nanofluids. Since, for specific values of $\Theta _\infty$ and $K_{nf}$, Rosseland absorptivity $k_r$ reduces while following a rise in values of Nr. This decrease in $k_r$ provides sufficient ground for enhancement of nonlinear thermal radiation flux $(\frac{\partial Q_r}{\partial y})$, which ensures that rate of radiative heat transfer to fluid grows rapidly. Consequently, the temperature profile of nanofluid rises.

**Figure 4:**
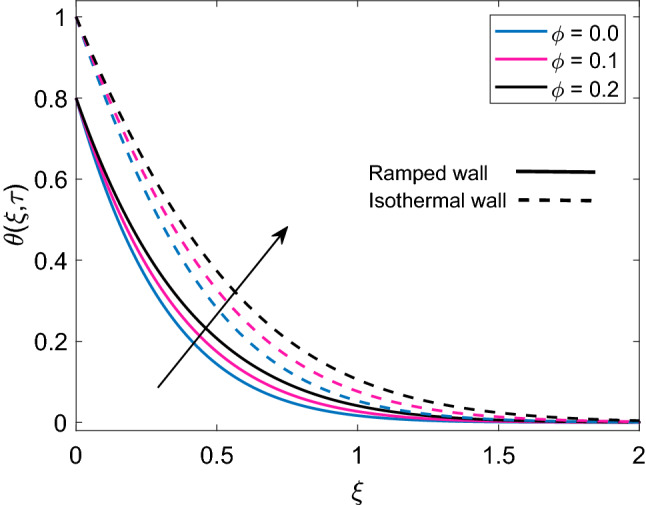
Temperature distribution [Eq. (31)] for various values of $\phi$ when $Nr=0.5$ and $Q=0.5$.

**Figure 5:**
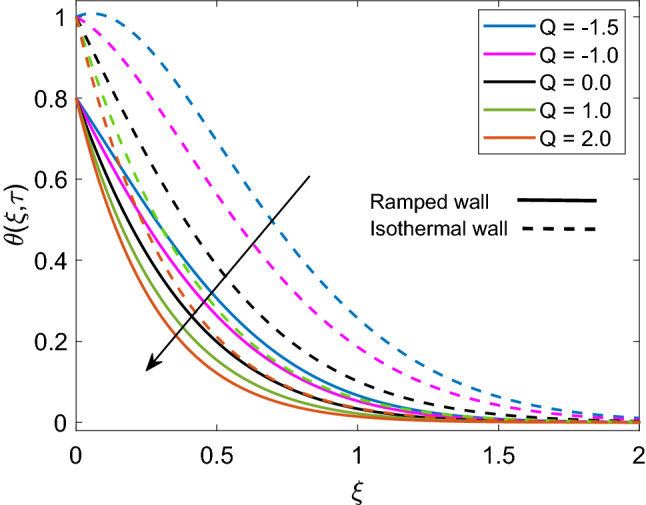
Temperature distribution [Eq. (31)] for various values of *Q* when $Nr=0.5$ and $\phi =0.1$.

**Figure 6:**
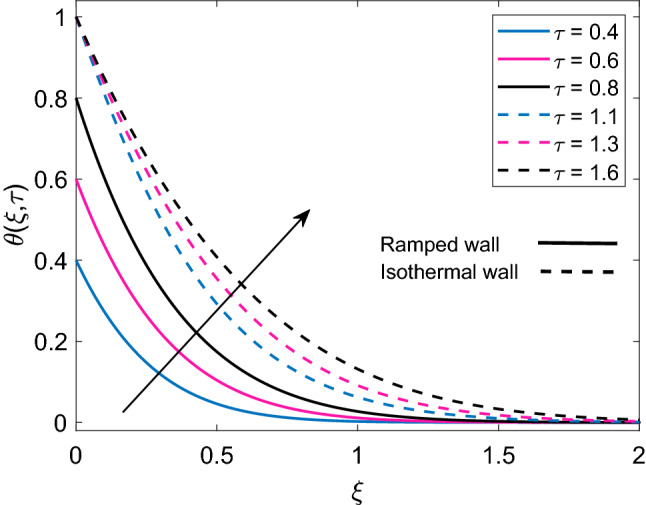
Temperature distribution [Eq. (31)] for various values of $\tau$ when $Nr=0.5$, $\phi =0.1$ and $Q=0.5$.

Figure 4 displays the crucial role of solid volume fraction $(\phi )$ of nanoparticles on temperature solution. It is found that enlargement in values of $\phi$ uplifts the temperature profile for both ramped and isothermal wall. Moreover dimensionless temperature of Cu-water nanofluid has thicker thermal boundary layer in contrast to pure base fluid water $(\phi =0)$. The physical justification of this higher thermal boundary layer is that suspension of Cu nanoparticles in water sums up the thermal conductivity of water and Cu and due to higher thermal conductivity of Cu, this addition results in increased thermal conductivity of nanofluid. This behavior reveals the significance of nanofluids in cooling and heating processes. Figure 5 demonstrates the temperature distribution, when a heat injector or sink (Q) is attached to the system. In respective graph, $Q>0$ denotes heat consumption, $Q<0$ denotes heat injection and $Q=0$ denotes that there is no heat injection or consumption. Physically, it is clear from the statement that heat injection means elevation of nanofluid temperature and heat consumption means nanofluid temperature faces a decay. This physical explanation well agrees with the results presented in corresponding Figure. Moreover, it is observed that temperature of nanofluid has lower profile in case of ramped wall boundary condition in presence of a heat source or sink. Transient effect on nanofluid temperature is sketched in Fig. 6. As $\tau$ progresses, temperature of nanofluid gets elevation for both ramped wall and isothermal wall. It is spotted that temperature has higher values for isothermal wall profile in contrast to ramped wall profile. Furthermore, initially nanofluid temperature has higher profile close to the wall and later far away from the wall it attains zero value asymptotically.

**Figure 7:**
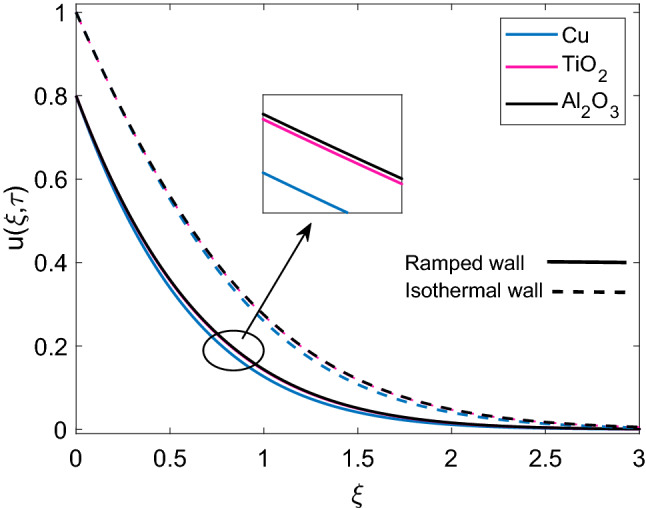
Comparison of velocity profile [Eq. (39)] of different nanofluids when $M=2.0$, $K=0.5$, $Gr=5.0$, $Nr=0.5$, $\phi =0.1$ and $Q=0.5$.

**Figure 8:**
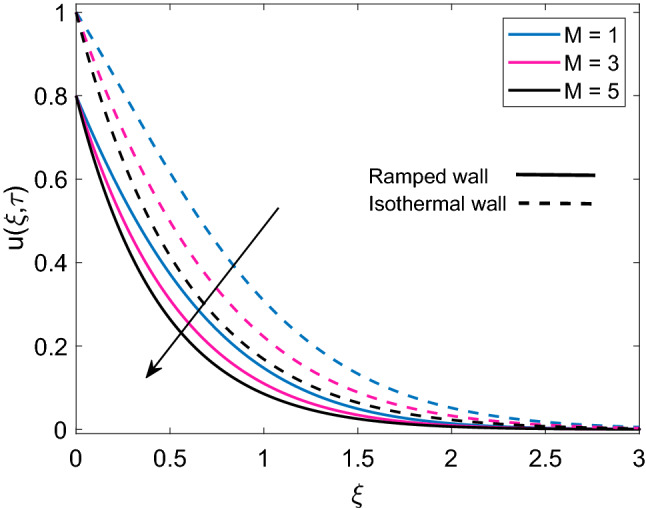
Velocity distribution [Eq. (39)] for various values of *M* when $K=0.5$, $Gr=5.0$, $Nr=0.5$, $\phi =0.1$ and $Q=0.5$.

**Figure 9:**
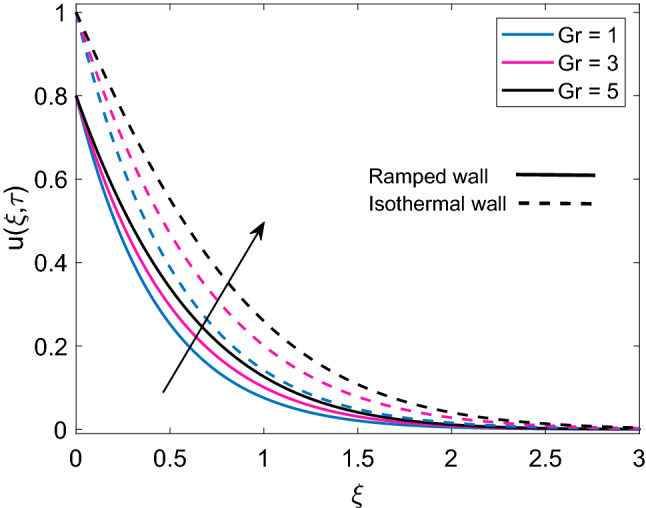
Velocity distribution [Eq. (39)] for various values of *Gr* when $M=2.0$, $K=0.5$, $Nr=0.5$, $\phi =0.1$ and $Q=0.5$.

Figure 7 describes the velocity profile of three different types of nanofluids (Cu-water, $\mathrm {Al_2O_3}$-water and $\mathrm {TiO_2}$-water) having same volume fraction. Due to higher density of Cu, nanofluid Cu-water has comparatively lower velocity than $\mathrm {TiO_2}$-water and $\mathrm {Al_2O_3}$-water. It is seen that velocity of $\mathrm {Al_2O_3}$-water and $\mathrm {TiO_2}$-water are very close to each other because of small density difference. Ramped wall velocity and isothermal wall velocity is compared and it is evaluated that nanofluid motion is more rapid in case of isothermal wall condition. The impact of magnetic parameter (M) on velocity distribution for ramped condition and constant condition is observed in Fig. 8. It is found that nanofluid velocity is decreasing function of M. This behavior is elucidated with the fact that imposition of magnetic field on an electrically insulated nanofluid acts as a source for generation of Lorentz force, which behaves as a viscous dragging force. Intensification of M enhances the dragging power of Lorentz force and eventually nanofluid comes to rest gradually. Figure 9 shows distribution of nanofluid velocity for variation in Grashof number (Gr). It is observed that velocity has a direct relation with Gr. The physical logic behind augmentation of velocity is strong thermal buoyancy force. Since Gr deals with viscous and buoyancy forces, rise in Gr leads to decrease the strength of viscous force. Consequently, close to the moving plate, nanofluid velocity is accelerated and as nanofluid flows far away from the plate, these flow favoring forces become weak and motion of nanofluid is gradually retarded to zero value. Moreover, velocity has higher profile for isothermal plate against ramped plate.

**Figure 10:**
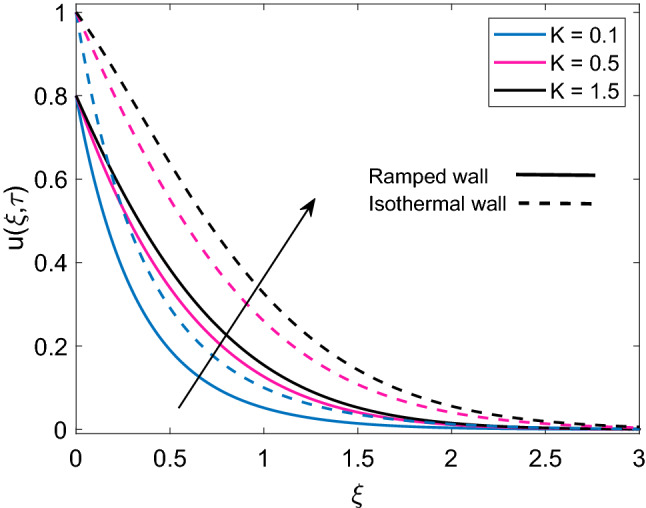
Velocity distribution [Eq. (39)] for various values of *K* when $M=2.0$, $Gr=5.0$, $Nr=0.5$, $\phi =0.1$ and $Q=0.5$.

**Figure 11:**
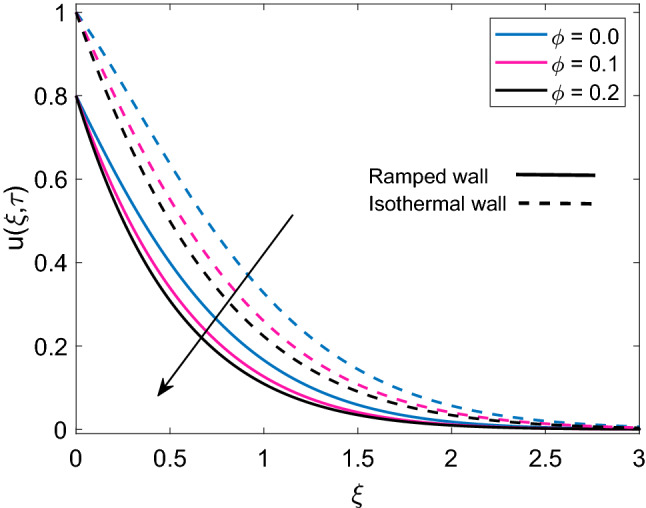
Velocity distribution [Eq. (39)] for various values of $\phi$ when $M=2.0$, $K=0.5$, $Gr=5.0$, $Nr=0.5$ and $Q=0.5$.

**Figure 12:**
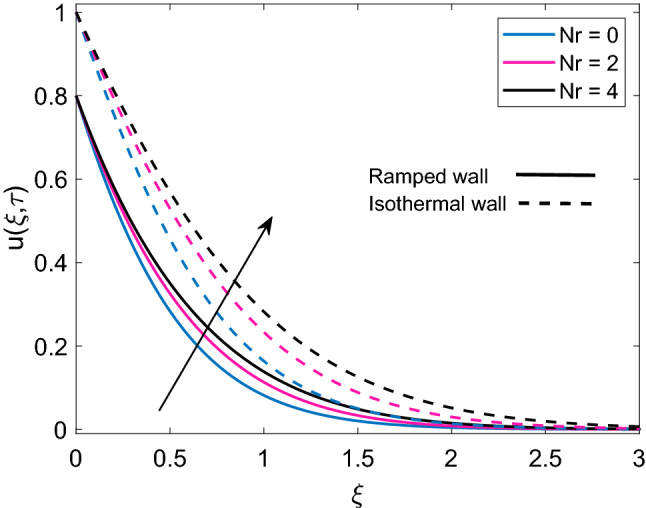
Velocity distribution [Eq. (39)] for various values of *Nr* when $M=2.0$, $K=0.5$, $Gr=5.0$, $\phi =0.1$ and $Q=0.5$.

**Figure 13:**
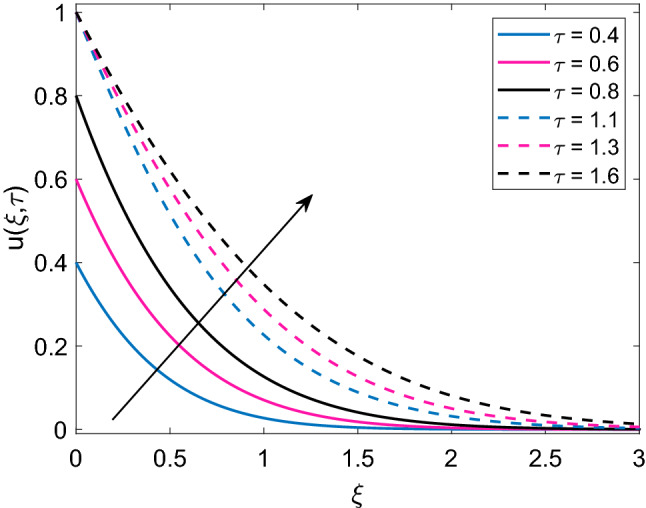
Velocity distribution [Eq. (39)] for various values of $\tau$ when $M=2.0$, $K=0.5$, $Gr=5.0$, $Nr=0.5$, $\phi =0.1$ and $Q=0.5$.

Figure 10 highlights the relationship between permeability parameter (K) and dimensionless velocity of nanofluid for both ramped wall boundary condition and isothermal wall boundary condition. It is spotted that increase in values of K accelerates nanofluid’s motion. Physically, it is justified by the reason that enrichment of permeability of mushy (porous) material pushes the viscous force to face a significant decay which results in an increment of the momentum development of the regime. In addition, velocity solution has lower values in case of ramped boundary conditions in contrast to constant boundary conditions. The effect of solid volume fraction $(\phi )$ of nanoparticles on dimensionless velocity is described in Fig. 11. It is seen that nanofluid velocity and $\phi$ share an inverse relation for the cases of ramped plate and isothermal plate. This is explained by the logic that an increase in $\phi$ means nanofluid has more density coming from the added fraction of nanoparticles, which reduces both momentum boundary layer thickness and velocity of nanofluid. Figure 12 depicts the influence of radiation parameter Nr on nanofluid velocity. A comparison is drawn for the isothermal plate velocity and ramped plate velocity and it is observed that for both types of plate, Nr has similar effects however, in case of isothermal plate, nanofluid velocity has higher profile. The argument behind augmentation of velocity due to increasing variation of Nr is increased rate of energy transfer. Bonds between nanofluid particles become weak due to this higher rate of energy transfer and ultimately it leads to reduce the power of viscous forces. The remaining weak viscous forces allow nanofluid to flow more rapidly. Figure 13 reveals the contribution of time $(\tau )$ in nanofluid flow. It is noted that nanofluid velocity enhances with an increase in $\tau$ for both the cases of ramped plate and isothermal plate.

**Figure 14:**
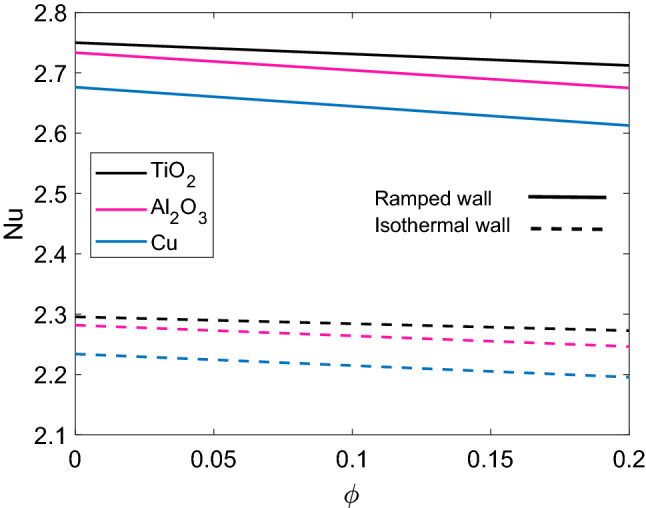
Comparison of heat transfer rate [Eq. (41)] for different nanofluids when $Nr=0.5$ and $Q=0.5$.

Rate of heat transfer at wall $\xi =0$ for different nanofluids is presented in Fig. 14. It is found that rate of heat transfer for Cu-water nanofluid is lowest among the three nanofluids. Higher thermal conductivity of Cu nanoparticles provides sufficient support to this behavior, as Cu-water nanofluid has a higher temperature, therefore the rate of heat transfer from plate to fluid is lower. The corresponding figure depicts that as $\phi$ increases rate of heat transfer decreases because enhancement in values of $\phi$ implies that temperature of fluid rises and ultimately small amount of heat is transferred from plate to the fluid. Furthermore, an interesting behavior is observed that rate of heat transfer has higher values in case of ramped plate. The contribution of several parameters in rate of heat transfer is computed in Table 1. It shows that rate of heat transfer has an inverse relation with $\tau$ and Nr, while heat consumption and heat injection increase and decrease the rate of heat transfer from plate to the fluid respectively.

**Table 1:** Nusselt number (rate of heat transfer) for variation of several parameters.

${\tau }$	Nr	Q	Nu
**0.3**	0.5	0.5	**1.5789**
**0.6**	–	–	**2.3298**
**0.9**	–	–	**2.9868**
0.6	**0.4**	–	**2.3961**
–	**0.6**	–	**2.2687**
–	**0.8**	–	**2.1596**
–	0.5	**− 0.6**	**1.8532**
–	–	**− 0.4**	**1.9452**
–	–	**0.0**	**2.4976**
–	–	**0.4**	**2.2888**
–	–	**0.6**	**2.3703**

The consecutive bold values of a parameter exhibit the variation in that parameter.

**Figure 15:**
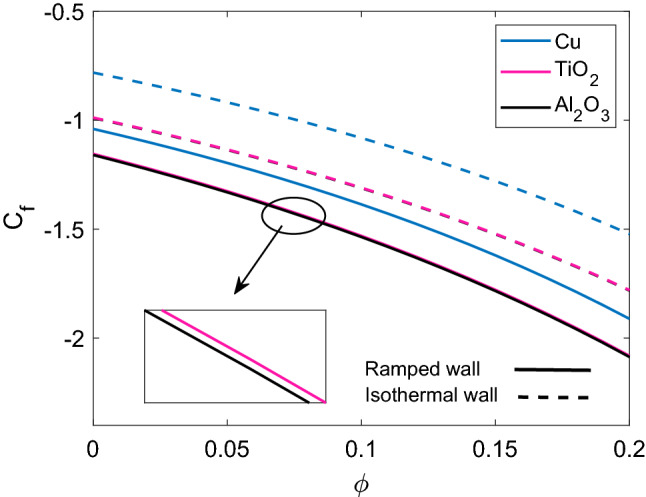
Comparison of skin friction [Eq. (43)] for different nanofluids when $M=2.0$, $K=0.5$, $Gr=5.0$, $Nr=0.5$ and $Q=0.5$.

For engineering process, skin friction (or shear stress) is a significant factor. In Fig. 15, the skin friction for different nanofluids is revealed for the cases of ramped wall and isothermal wall. It is observed that shear stress at wall $\xi =0$ is greater for Cu-water due to higher density of Cu nanoparticles. Moreover, $\mathrm {Al_2O_3}$ and $\mathrm {TiO_2}$ have almost the same shear stress because their densities are very close to each other. Shear stress for isothermal plate is found to have higher curves as compared to ramped plate. Table 2 provides the numerical computations of skin friction for variation of influencing parameters. It is evaluated that skin friction is decreasing function of M, $\phi$ and $\tau$, while Gr, K and Nr bring an increase in the value of skin friction at wall. All the numerical values used to draw graphs and prepare tables are given in Table 3.

**Table 2:** Skin friction (shear stress) for variation of several parameters.

${\tau }$	M	K	${\phi }$	Gr	Nr	${{C}}_{{{f}}}$
**0.3**	2.0	0.5	0.1	5.0	3.0	**− 0.9710**
**0.6**	–	–	–	–	–	**− 1.4069**
**0.9**	–	–	–	–	–	**− 1.7779**
0.6	**1.0**	–	–	–	–	**− 1.2333**
–	**2.0**	–	–	–	–	**− 1.4069**
–	**3.0**	–	–	–	–	**− 1.5694**
–	2.0	**0.1**	–	–	–	**− 2.4755**
–	–	**0.5**	–	–	–	**− 1.4874**
–	–	**0.9**	–	–	–	**− 1.2570**
–	–	0.5	**0.00**	–	–	**− 0.8136**
–	–	–	**0.02**	–	–	**− 0.9255**
–	–	–	**0.04**	–	–	**− 1.0402**
–	–	–	0.1	**1.0**	–	**− 1.8882**
–	–	–	–	**3.0**	–	**− 1.6475**
–	–	–	–	**4.0**	–	**− 1.5272**
–	–	–	–	5.0	**1.0**	**− 1.4904**
–	–	–	–	–	**3.0**	**− 1.4069**
–	–	–	–	–	**5.0**	**− 1.3568**

The consecutive bold values of a parameter exhibit the variation in that parameter.

**Table 3:** Thermophysical features of water and nanoparticles^60^.

Fluid/nanoparticles	$\rho (\frac{kg}{m^3})$	$C_p(\frac{J}{kg K})$	$k(\frac{W}{m K})$	$\beta \times 10^5 (\frac{1}{K})$	$\sigma$ $(\frac{S}{m})$
Water	997.1	4179	0.613	21	$5.5\times 10^{-6}$
Copper (Cu)	8933	385	401	1.67	$59.6\times 10^6$
Alumina $({{Al}_{{2}}{} {O}_{{3}}})$	3970	765	40	0.85	$35\times 10^6$
Titanium oxide $({{TiO}_{{2}}})$	4250	686.2	8.9538	0.90	$2.6\times 10^6$

## Conclusion

The prime concern of this investigation is to evaluate the physical effects of application of simultaneous ramped wall velocity and ramped wall temperature condition on unsteady, MHD convection flow of some nanofluids over an infinite vertical plate. The Darcy’s law is applied to encounter the porosity of the medium. In addition, heat injection/consumption and heat radiative flux are also inculcated in the model. It is worth mentioning that simultaneous application of ramped and isothermal wall boundary conditions is physically significant but analytical handling of resulted mathematical expressions is burdensome at the same time. However, in this work, exact solutions are derived by employing the Laplace transform and presented in close form. The impact of associated parameters on dimensionless temperature and velocity solutions are illustrated graphically, meanwhile, the computed results for skin friction (shear stress) and Nusselt number are provided through tables. The solutions for isothermal plate boundary condition and ramped plate boundary condition are also compared.

The principal investigations of this analysis are concluded as

- In case of temperature, velocity, shear stress and rate of heat transfer, respective profile behaves in a similar manner for both ramped wall and isothermal wall boundary conditions.
- Momentum and thermal boundary layers have more thickness in case of isothermal wall in contrast to ramped wall.
- Nanofluid velocity is a decreasing function of magnetic parameter M and volume fraction $\phi$.
- Momentum boundary layer thickness increases for increasing values of Grashof number Gr, permeability parameter K and radiation parameter Nr.
- The temperature of Cu-water is found to be higher but an exactly inverse statement holds for velocity field.
- Cu-water has higher skin friction at the wall (associated to shear stress).
- Rate of heat transfer at the wall is found to be higher for $\mathrm {TiO_2}$-water (related to Nusselt number).

## Supplementary information

Supplementary Information.

## Data Availability

All the relevant material is available.

## Nomenclature

${\mathbf {V}}$: Fluid velocity vector
$${\mathbf {J}}$$: Current density
${\mathbf {B}}$: Total magnetic field
${\mathbf {r}}$: Darcy resistant vector
$$\rho$$: Fluid density
*t*: time
*g*: Force of gravity
$\beta$: Thermal expansion coefficient
$$\Theta$$: Fluid temperature
$$\Theta _\infty$$: Ambient temperature
$K_f$: Thermal conductivity of fluid
$\gamma _1$: Viscous dissipation term
$C_p$: Specific heat capacitance
$Q_r$: Thermal radiation flux
*Q*: Heat injection/consumption constant
$$k^*$$: Porosity term
E: Electric field
$B_0$: Imposed magnetic field
$$t_0$$: Characteristic time
$\phi$: Nanoparticle volume fraction
$k_r$: Coefficient of Rosseland adsorption
*u*: Dimensionless velocity
$$y,\xi$$: Space variables
Gr: Grashof number
M: Magnetic parameter
Nr: Radiation parameter
Pr: Prandtl number
K: Permeability parameter
$Q_0$: Heat injection/consumption parameter
${\mathcal {L}}$: Laplace transform operator
${\mathcal {L}}^{-1}$: Laplace transform operator
*p*: Complex Laplace frequency
G(.): Standard Heaviside function
Nu: Local Nusselt number
$$C_f$$: Skin friction
${\text {erfc}}(.)$: Complementary error function
${\text {erf}}(.)$: Standard error function
$$\gamma _2$$: Permeability
$$\mu _m$$: Magnetic permeability
$\sigma$: Fluid electrical permeability
$$U_0$$: Uniform velocity
$$\Theta _w$$: Wall temperature
$\sigma _r$: Stefan–Boltzmann constant
$\nu _w$: Fluid kinematic viscosity
$$\tau$$: Dimensionless time
