# Supplementary material for: An exact analysis of unsteady MHD free convection flow of some nanofluids with ramped wall velocity and ramped wall temperature accounting heat radiation and injection/consumption
Source: Sci Rep. 2020 Oct 20;10:17830. doi: 10.1038/s41598-020-74739-w (PMC7575599; doi:10.1038/s41598-020-74739-w)
Supplement: Supplementary file 1 — Supplementary Information. [file 41598_2020_74739_MOESM1_ESM.pdf]

An exact analysis of unsteady MHD ree convection flow of some nanofluids with ramped wall velocity and ramped wall temperature accounting heat radiation and injection/consumption

Talha Anwar [1], Poom Kumam[2,3,4,†], Wiboonsak Watthayu [1]

[1] Department of Mathematics, Faculty of science, King Mongkut's University of Technology Thonburi (KMUTT), 126 Pracha-Uthit Road, Bang Mod, Thung Khru, Bangkok 10140, Thailand.
[2] KMUTTFixed Point Research Laboratory, Room SCL 802 Fixed Point Laboratory, Science Laboratory Building, Department of Mathematics, Faculty of Science, King Mongkut's University of Technology Thonburi (KMUTT), 126 Pracha-Uthit Road, Bang Mod, Thung Khru, Bangkok 10140, Thailand.
[3] Center of Excellence in Theoretical and Computational Science (TaCS-CoE), Science Laboratory Building, King Mongkut's University of Technology Thonburi (KMUTT), 126 Pracha-Uthit Road, Bang Mod, Thung Khru, Bangkok 10140, Thailand.
[4] Department of Medical Research, China Medical University Hospital, China Medical University, Taichung 40402, Taiwan.

[†] Corresponding author: poom.kumam@mail.kmutt.ac.th (P. Kumam)

1. Appendix

$$\frac{\partial \psi_1}{\partial \xi}\Big|_{\xi=0} = -\sqrt{\frac{\alpha\tau}{\pi}} e^{-\frac{\lambda\tau}{\alpha}} - \operatorname{erf}\left(\sqrt{\frac{\lambda\tau}{\alpha}}\right)\left[\frac{\alpha}{2\sqrt{\lambda}} + \sqrt{\lambda\tau}\right]. \tag{A.1}$$

$$\frac{\partial \hat{\psi}_1}{\partial \xi}\Big|_{\xi=0} = -\sqrt{\frac{\alpha(\tau-1)}{\pi}} e^{-\frac{\lambda(\tau-1)}{\alpha}} - \operatorname{erf}\left(\sqrt{\frac{\lambda(\tau-1)}{\alpha}}\right)\left[\frac{\alpha}{2\sqrt{\lambda}} + \sqrt{\lambda(\tau-1)}\right]. \tag{A.2}$$

$$\frac{\partial \psi_2}{\partial \xi}\Big|_{\xi=0} = -\sqrt{\frac{\eta\tau}{\pi}} e^{-\frac{\omega\tau}{\eta}} - \operatorname{erf}\left(\sqrt{\frac{\omega\tau}{\eta}}\right)\left[\sqrt{\omega\tau} + \frac{\eta}{2\sqrt{\omega}}\right],$$

$$\frac{\partial \hat{\psi}_2}{\partial \xi}\Big|_{\xi=0} = -\sqrt{\frac{\eta(\tau-1)}{\pi}} e^{-\frac{\omega(\tau-1)}{\eta}} - \operatorname{erf}\left(\sqrt{\frac{\omega(\tau-1)}{\eta}}\right)\left[\sqrt{\omega(\tau-1)} + \frac{\eta}{2\sqrt{\omega}}\right]. \tag{A.3}$$

$$\frac{\partial E_1}{\partial \xi}\Big|_{\xi=0} = -\frac{2\eta}{\sqrt{\pi\tau}} e^{-(b+\eta\omega)\tau} - 2(\sqrt{\omega+b\eta})\operatorname{erf}\left(\sqrt{(b+\omega\eta)\tau}\right),$$

$$\frac{\partial E_2}{\partial \xi}\Big|_{\xi=0} = -\frac{2\alpha}{\sqrt{\pi\tau}} e^{-(b+\alpha\lambda)\tau} - 2(\sqrt{\lambda+b\alpha})\operatorname{erf}\left(\sqrt{(b+\lambda\alpha)\tau}\right),$$

$$\frac{\partial \hat{E}_1}{\partial \xi}\Big|_{\xi=0} = -\frac{2\eta}{\sqrt{\pi(\tau-1)}} e^{-(b+\eta\omega)(\tau-1)} - 2(\sqrt{\omega+b\eta})\operatorname{erf}\left(\sqrt{(b+\omega\eta)(\tau-1)}\right),$$

$$\frac{\partial \hat{E}_2}{\partial \xi}\Big|_{\xi=0} = -\frac{2\alpha}{\sqrt{\pi(\tau-1)}} e^{-(b+\alpha\lambda)(\tau-1)} - 2(\sqrt{\lambda+b\alpha})\operatorname{erf}\left(\sqrt{(b+\lambda\alpha)(\tau-1)}\right). \tag{A.4}$$

$$\frac{\partial F_1}{\partial \xi}\Big|_{\xi=0} = -\frac{2}{\sqrt{\pi}}\left(\tau+\frac{1}{b}\right)\sqrt{\frac{\eta}{\tau}} e^{-\frac{\omega\tau}{\eta}} - \operatorname{erf}\left(\sqrt{\frac{\omega\tau}{\eta}}\right)\left[\frac{\eta}{\sqrt{\omega}} + 2\sqrt{\omega}\left(\tau+\frac{1}{b}\right)\right],$$

$$\frac{\partial F_2}{\partial \xi}\Big|_{\xi=0} = -\frac{2}{\sqrt{\pi}}\left(\tau + \frac{1}{b}\right)\sqrt{\frac{\alpha}{\tau}}e^{-\frac{\lambda\tau}{\alpha}} - \operatorname{erf}\left(\sqrt{\frac{\lambda\tau}{\alpha}}\right)\left[\frac{\alpha}{\sqrt{\lambda}} + 2\sqrt{\lambda}\left(\tau + \frac{1}{b}\right)\right],$$

$$\frac{\partial \hat{F}_1}{\partial \xi}\Big|_{\xi=0} = -\frac{2}{\sqrt{\pi}}\left(\tau - 1 + \frac{1}{b}\right)\sqrt{\frac{\eta}{\tau-1}}e^{-\frac{\omega(\tau-1)}{\eta}} -$$
$$\operatorname{erf}\left(\sqrt{\frac{\omega(\tau-1)}{\eta}}\right)\left[\frac{\eta}{\sqrt{\omega}} + 2\sqrt{\omega}\left(\tau - 1 + \frac{1}{b}\right)\right],$$

$$\frac{\partial \hat{F}_2}{\partial \xi}\Big|_{\xi=0} = -\frac{2}{\sqrt{\pi}}\left(\tau - 1 + \frac{1}{b}\right)\sqrt{\frac{\alpha}{\tau-1}}e^{-\frac{\lambda(\tau-1)}{\alpha}} -$$
$$\operatorname{erf}\left(\sqrt{\frac{\lambda(\tau-1)}{\alpha}}\right)\left[\frac{\alpha}{\sqrt{\lambda}} + 2\sqrt{\lambda}\left(\tau - 1 + \frac{1}{b}\right)\right]. \qquad \text{(A.5)}$$
